# Efficacy analysis of minimally invasive surgery for Raynaud’s syndrome

**DOI:** 10.1186/s12893-023-02225-x

**Published:** 2023-10-14

**Authors:** Fengwei Yu, Yongtao Liu, Chengnian Zhang, Botao Pang, Daijie Zhang, Wei Zhao, Xuecheng Li, Weiqiang Yang

**Affiliations:** 1 Hand Microsurgery, Binzhou Medical University Hospital, Binzhou, 256600 China; 2 The First Clinical School of Binzhou Medical University, Binzhou, 256600 China

**Keywords:** Adventitial release, Hand, Microsurgery, Raynaud’s syndrome, Surgical treatment, Sympathectomy

## Abstract

**Background:**

Raynaud’s syndrome (RS), also referred to as Raynaud’s phenomenon, is a vasospastic disorder causing episodic color changes in extremities upon exposure to cold or stress. These manifestations, either primary Raynaud’s phenomenon (PRP) or associated with connective tissue diseases like systemic sclerosis (SSc) as secondary Raynaud’s phenomenon (SRP), affect the quality of life. Current treatments range from calcium channel blockers to innovative surgical interventions, with evolving efficacy and safety profiles.

**Methods:**

In this retrospective study, patients diagnosed with RS were selected based on complete medical records, ensuring homogeneity between groups. Surgeries involved microscopic excision of sympathetic nerve fibers and stripping of the digital artery’s adventitia. Postoperative care included antibiotics, analgesia, oral nifedipine, and heat therapies. Evaluation metrics such as the VAS pain score and RCS score were collected bi-weekly. Data analysis was conducted using SPSS 26.0, with significance set at p < 0.05.

**Results:**

In total, 15 patients formed the experimental group, with five presenting fingertip soft tissue necrosis and ten showing RS symptoms. Comparative analysis of demographic data between experimental and control groups, both containing 15 participants, demonstrated no significant age and gender difference. However, the “Mean Duration of RP attack” in the experimental group was notably shorter (9.47 min ± 0.31) than the control group (19.33 min ± 1.79). The RS Severity Score also indicated milder severity for the experimental cohort (score: 8.55) compared to the control (score: 11.23). Postoperative assessments at 2, 4, and 6 weeks revealed improved VAS pain scores, RCS scores, and other measures for the experimental group, showing significant differences (p < 0.05). One distinctive case showcased a variation in the common digital nerve and artery course in an RS patient.

**Conclusion:**

Our retrospective analysis on RS patients indicates that microsurgical techniques are safe and effective in the short term. As surgical practices lean towards minimally invasive methods, our data supports this shift. However, extensive, prospective studies are essential for conclusive insights.

## Introduction

Raynaud’s syndrome (RS), often termed as Raynaud’s phenomenon or Raynaud’s disease, epitomizes a complex vasospastic disorder eliciting episodic color shifts (pallor, cyanosis, and erythema) in extremities upon encountering cold or emotional stimuli. Underpinning its pathophysiology is an augmented vasoconstrictive reaction coupled with hindered vasodilation. Such manifestations can either be idiopathic, recognized as primary Raynaud’s phenomenon (PRP), or be concomitant with underlying connective tissue diseases (CTD) such as systemic sclerosis (SSc), denoted as secondary Raynaud’s phenomenon (SRP). A study distinguishes between PRP and SRP by emphasizing the importance of morphological and functional diagnostic tools, where PRP stands benign, while SRP correlates with specific CTD patterns, notably SSc. In particular, nailfold videocapillaroscopy (NVC) and laser tools have been instrumental in such differentiations. Another research accentuates that while PRP is prevalent, affecting 3-5% of the general populace, SRP, especially linked to systemic sclerosis, remains rare but can escalate to severe conditions like digital ulceration. Regardless of its type, the syndrome considerably diminishes the quality of life. As RS continues to perplex the medical community, current strides, inclusive of microsurgical techniques, are broadening therapeutic horizons, though challenges persist for comprehensive understanding and management [1, 2]. The total ischemic time lasts 15–20 min [3]. RS, in its primary form, often involves the index, middle, and ring fingers, typically sparing the thumb. However, in secondary forms associated with scleroderma vasculopathy, the thumb is not spared from involvement, indicating severe nutritive vessels’ microcirculation impairment [4, 5, 4, 5]. Currently, calcium channel blockers (e.g., nifedipine) are recommended as the first-line treatment for RS, but other drugs, such as phosphodiesterase-5 (PDE5) inhibitors, are also given in severe cases [6]. However, in the past years, surgical interventions have been used only for refractory RS and are usually considered in Sjögren’s syndrome and digital ulcers or necrosis cases. In light of this, our study aims to delve into the efficacy and safety of newer interventions, specifically digital sympathectomy and botulinum toxin injections, as potential treatment modalities for RS. Because of uncertain long-term efficacy and several complications, surgical interventions such as cervical sympathectomy are no longer performed. Moreover, in recent years, numerous studies have used digital (not cervical/lumbar) sympathectomy and botulinum toxin injections for treating RS with promising results [7–9].

## Methods

Patients for the retrospective study were selected based on the availability of complete medical records and their diagnosis of Raynaud’s syndrome. Basic demographic and clinical data of both groups, including age, sex, duration of symptoms, and severity of RS, were collected. The selection aimed to maintain comparable baseline characteristics between the two groups. This was done to minimize potential biases. We ensured that both groups were homogenous concerning major demographic and clinical factors.

### Surgical procedures

After successful anesthesia, the patients were placed in the supine position, and the affected limb was abducted to 90°. After routine upper limb disinfection, a sterile towel drape was applied, and a pneumatic tourniquet was used for the upper limbs (depending on the patient’s blood pressure). The incision was extended from the proximal end of the affected finger, toward the course of the common digital artery and the nerve. After reflecting skin and subcutaneous tissue, the palmar aponeurosis was incised, and the common digital artery and corresponding nerve were exposed microscopically. After separating the common digital nerve, the sympathetic nerve fibers innervating the common digital artery were excised under the microscope. The digital artery was stripped of adventitia for 0.5–1 cm, every 1–2 cm after its separation. A tourniquet was applied to ensure the sensitive capillary reaction of each finger, and the surgical incisions were wet-compressed with warm saline. After careful hemostasis, suturing was done, followed by the application of compressive dressings.

### Postoperative treatment

All patients received postoperative antibiotics for 24 h, followed by patient-controlled analgesia and oral nifedipine. The patients with fingertip necrosis underwent debridement in the second stage. All patients (control and experimental groups) were treated with heat and lamp therapies to maintain an ambient temperature of 26 °C. The patients were given behavioral interventions before discharge and were instructed to pay attention to keeping their digits warm and avoiding cold exposure. Although there was little evidence demonstrating that daily lifestyle habits influenced RS pathogenesis [10, 11], patients should be encouraged to quit smoking to promote vasoconstriction [10].

### Evaluation methods

During daily examinations before discharge, the VAS pain score, RCS score, Quick-DASH scale score, digital ulcer score, and cold water stimulation test results were collected. Post-discharge, the patients reported to the outpatient department for reexamination every 2 weeks, and relevant conditions were statistically analyzed.

### Statistical analysis

SPSS 26 0.0 software (IBM, Armonk, NY, USA) was used for data analysis. Before undertaking the main statistical analysis, the normality of the data distribution was assessed using the Shapiro-Wilk test. Enumeration data were compared using the χ^2^ test, while the measurement data were expressed as mean ± standard deviation. Furthermore, the t-test was used for intergroup comparisons. *p* < 0.05 was considered statistically significant.

## Results

Of the fifteen experimental group patients, five had fingertip soft tissue necrosis without any phalangeal exposure or infection. The other ten presented with symptoms of Raynaud’s syndrome, including discoloration, pain, and digital ulcers but did not have necrotic changes.

In our study, we meticulously compared the demographic and clinical parameters between the experimental and control groups. From a demographic perspective, both cohorts consisted of 15 participants. The experimental group’s age distribution was centered at a mean of 49.0 years with a standard deviation of 12.2 years, whereas the control group presented a closely aligned mean age of 49.3 years with a standard deviation of 13.9 years. Gender distribution differed marginally with the experimental group comprising 40% males and 60% females, while the control group contained a slightly higher male proportion of 53.3% and a female proportion of 46.7%. In terms of clinical observations, the average duration of symptoms, inferred as the “Mean Duration of RP attack”, was distinctly shorter in the experimental group at *9.47 min (± 0.31 min)* compared to the control group’s longer average of *19.33 min (± 1.79 min)*. Furthermore, the RS Severity Score, which is an amalgamation of scores from various tests like the Cold water stimulation test, fingertip ulcer score, RCS Average attack time, RCS Number of episodes per week, and VAS, showed a milder severity in the experimental group (score: *8.55*) in contrast to a more pronounced severity in the control group (score: *11.23*). These findings offer crucial insights into the differing clinical presentations and demographic distributions between the two groups, laying a foundation for further exploration and interventions (Table 1).

**Table 1:** Demographic and Clinical Characteristics of the Study Population

Variable	Experimental Group	Control Group
Demographics		
Number of Participants	15	15
Age (mean ± SD)	49.0 ± 12.2 years	49.3 ± 13.9 years
Male, n (%)	6 (40%)	8 (53.3%)
Female, n (%)	9 (60%)	7 (46.7%)
Clinical Data		
Duration of Symptoms (mean ± SD)	9.47 ± 0.31 min	19.33 ± 1.79 min
RS Severity Score (mean ± SD)	8.55	11.23

During the postoperative follow-up at 2, 4, and 6 weeks, no surgical complications were observed. The VAS pain scores, RCS scores, Quick-DASH scale scores, digital ulcer scores, and cold water stimulation test results of the experimental group were superior to the control group patients with *statistically significant differences (p < 0.05)*. However, no significant differences were observed in the sex and age between the treatment and the control groups (*p* > 0.05, Tables 2 and 3.

**Table 2:** Distribution of postoperative follow-up of all the patients at 2, 4, and 6 weeks

Group	Cases	VAS pain score			Quick-DASH scale score			digital ulcer score	cold water stimulation test (fingertip temperature at corresponding time)
		2 weeks post-op	4 weeks post-op	6 weeks post-op	2 weeks post-op	4 weeks post-op	6 weeks post-op		δT
Experimental group	15	2.01 ±1.58	2.00 ±1.60	2.07 ±1.58	16.5 ±17.3	16.8 ±16.5	15.3 ±18.8	1.5 ±1.2	18.6 ±6.0
Control group	15	4.53 ±1.96	5.00 ±1.77	4.53 ±1.96	43.8 ±21.9	52.9 ±22.8	57.4 ±24.2	2.7 ±1.0	13.8 ±4.8
T value		-3.796	-4.861	-4.564	-3.783	-4.964	-5.331	-2.621 (Z value)	2.386
P value		0.01	0.00	0.00	0.01	0.00	0.00	0.009	0.024

Note: Both hands were put into 4 °C ice water for 20 s, then removed and quickly dried (the patients were instructed not to rub their hands), and the skin temperature of each fingertip of the second to fourth fingers of both the hands was immediately measured and recorded as T0; the fingers did not touch any object, and naturally recovered at room temperature for 3 min, then re-measured and recorded the skin temperature of each fingertip, which was recorded as T3; T3-T0 of each finger of each hand was calculated, respectively, and the mean was obtained, which was recorded as δT

**Table 3:** Distribution of RS attacks influenced by RCS score, age, and sex in both the study groups

Results	Cases	Mean Duration of RP attack (min)			The average number of RP attacks per week			Sex		Age (years)
		2 weeks	4 weeks	6 weeks	0–2 weeks	3–4 weeks	5–6 weeks	Male	Female	
Experimental group	15	9.2 ± 5.0	9.4 ± 4.8	9.8 ± 4.8	11.4 ± 5.7	10.5 ± 4.5	9.7 ± 4.9	6	9	49.0 ± 13.0
Control group	15	18.3 ± 12.8	18.3 ± 12.6	21.4 ± 19.8	16.8 ± 9.2	17.4 ± 9.4	17.1 ± 9.5	8	7	49.3 ± 13.6
T value (or Z value)		-2.954 (Z value)	-3.239 (Z value)	-3.294 (Z value)	-2.730 (Z value)	-3.051 (Z value)	-2.662	-0.714		-0.55
P value		0.003	0.001	0.001	0.006	0.002	0.013	0.481		0.957

### A case description

**Fig. 1:**
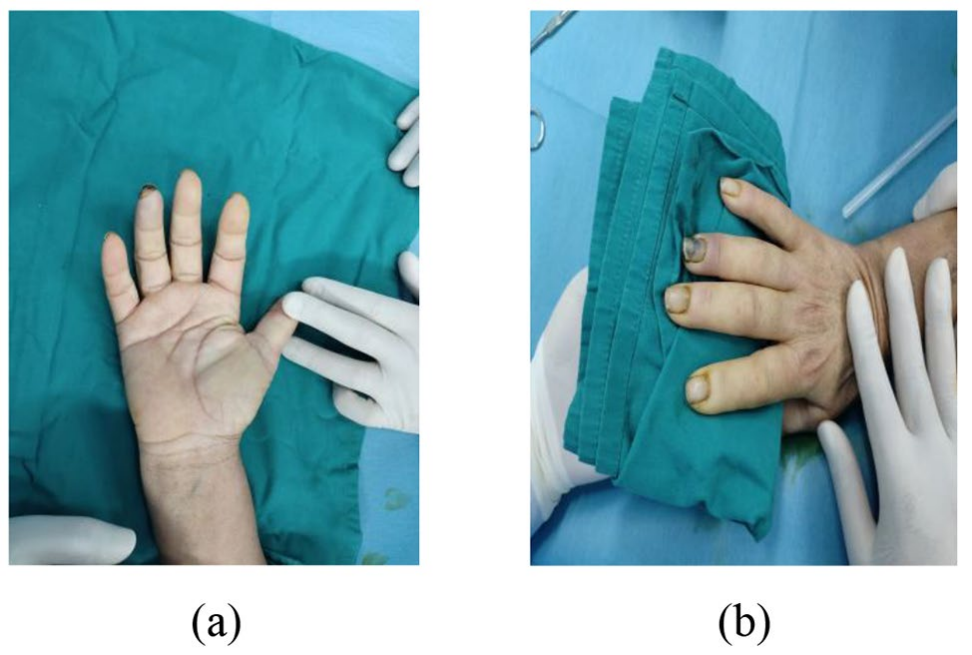
Preoperative exterior image

**Fig. 2:**
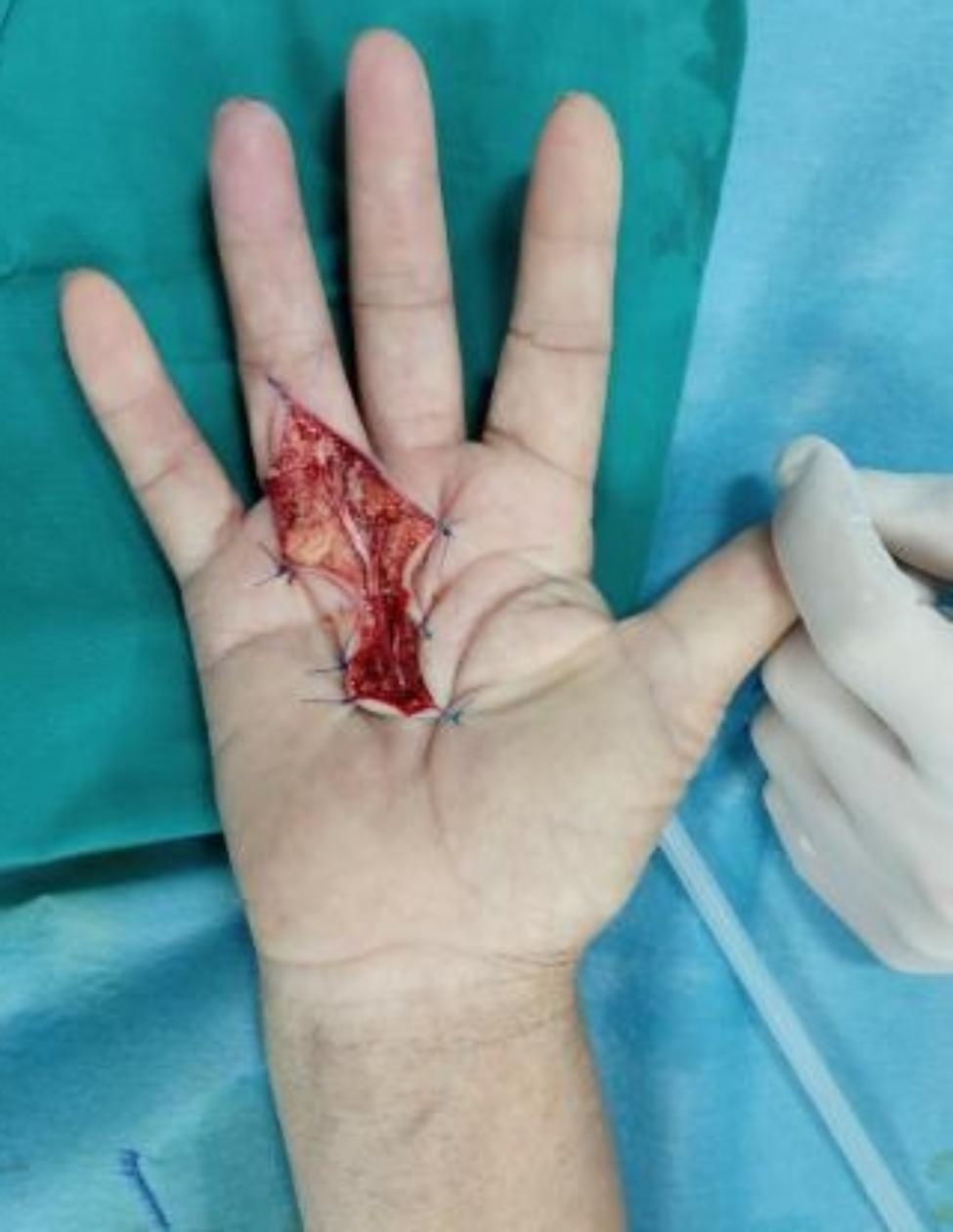
Surgical incision

**Fig. 3:**
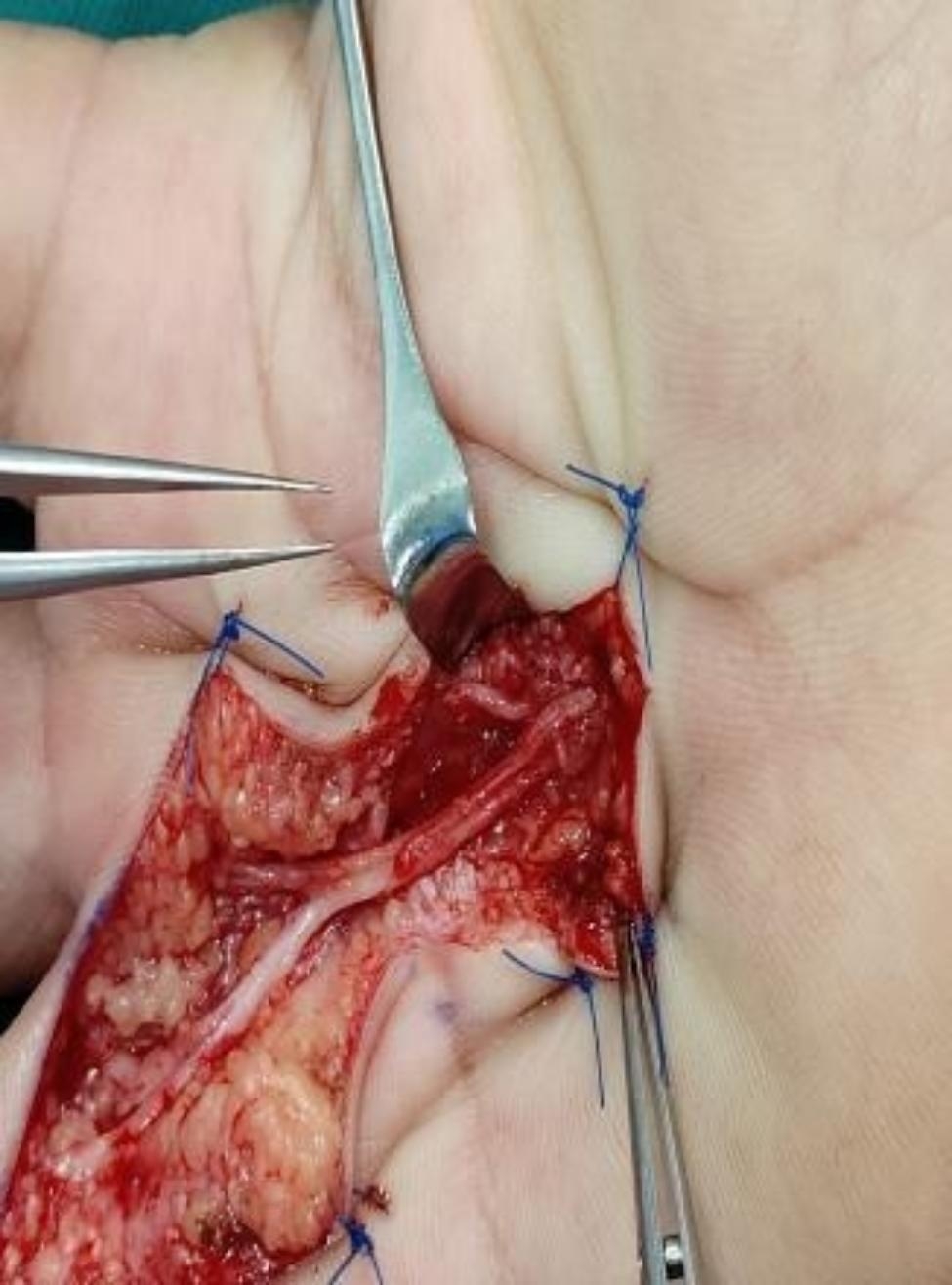
Visible variation in the course of vascular nerve bundles

**Fig. 4:**
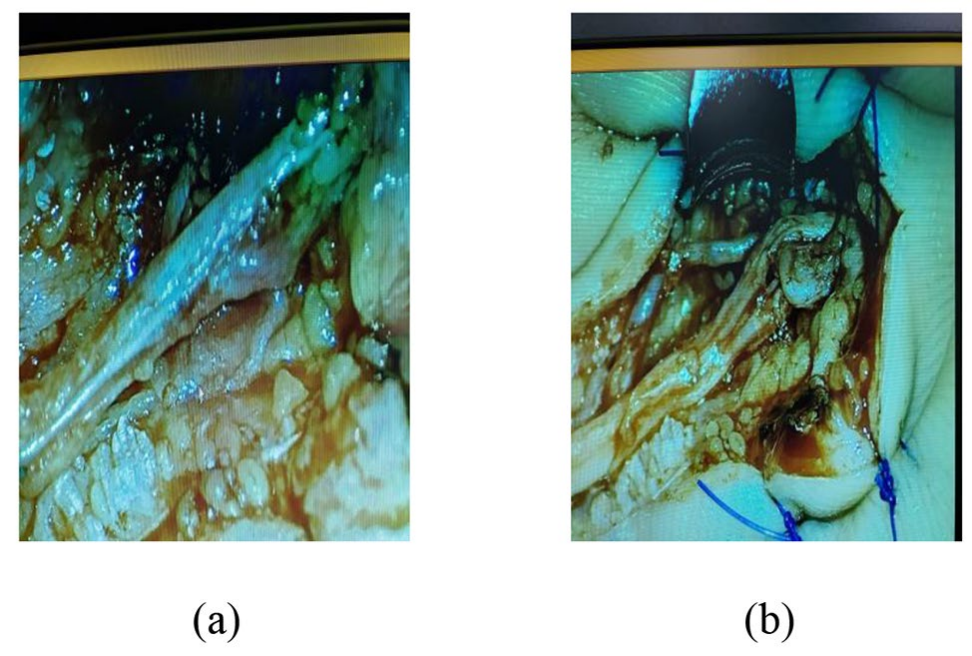
Variation in the course of vascular nerve bundles under the microscope

**Fig. 5:**
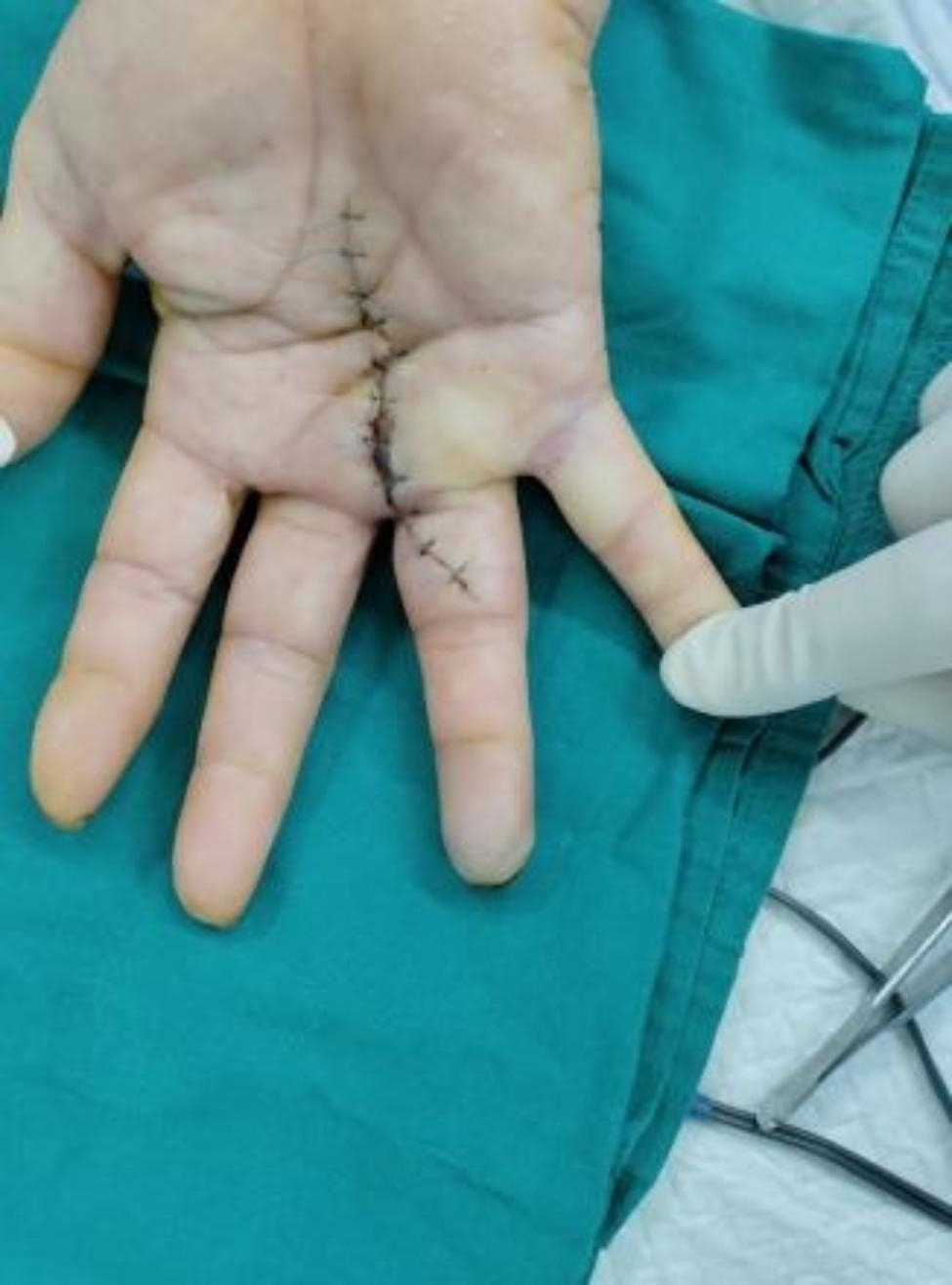
Postoperative follow-up results after 2 weeks

**Fig. 6:**
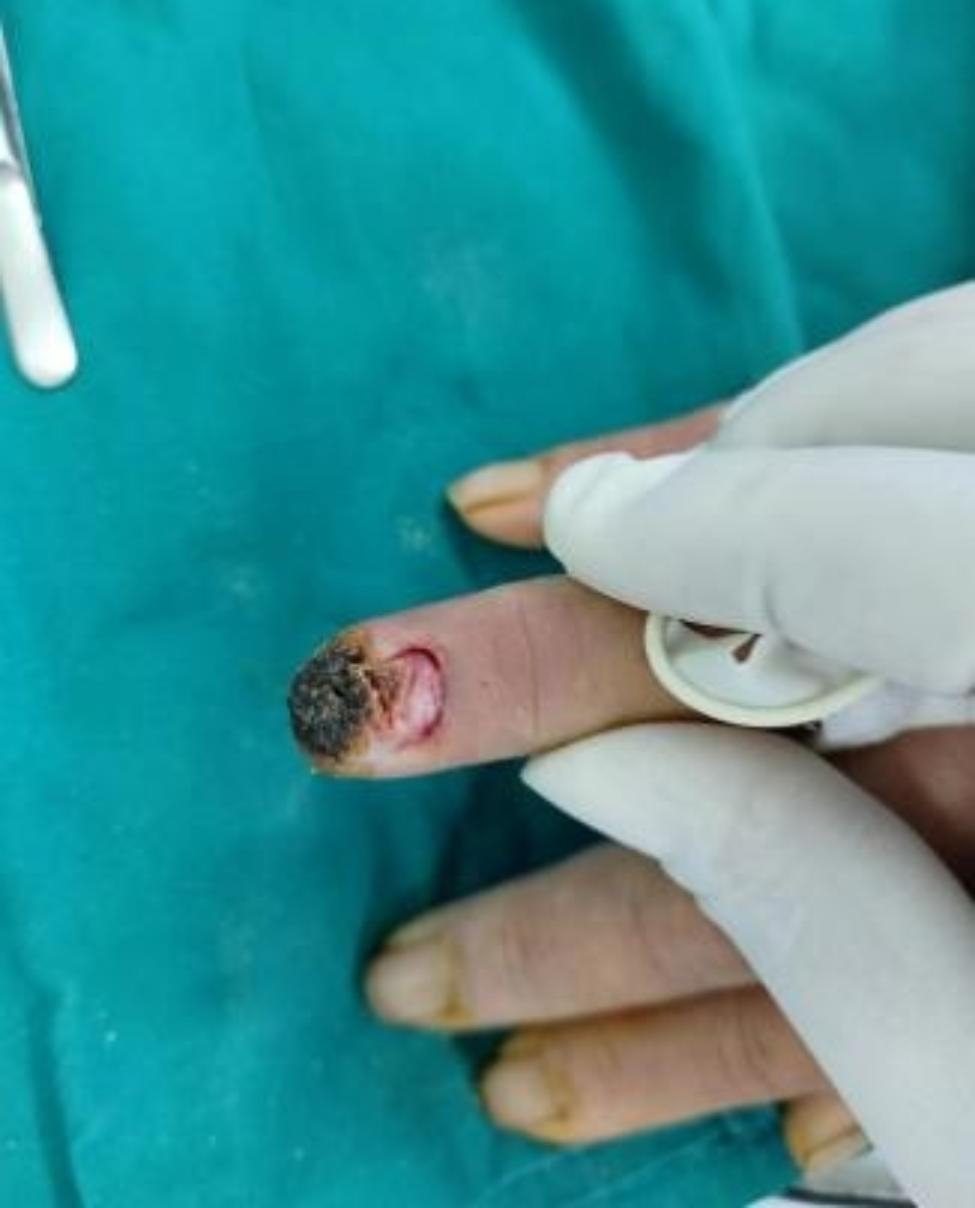
No enlargement of fingertip necrosis

**Fig. 7:**
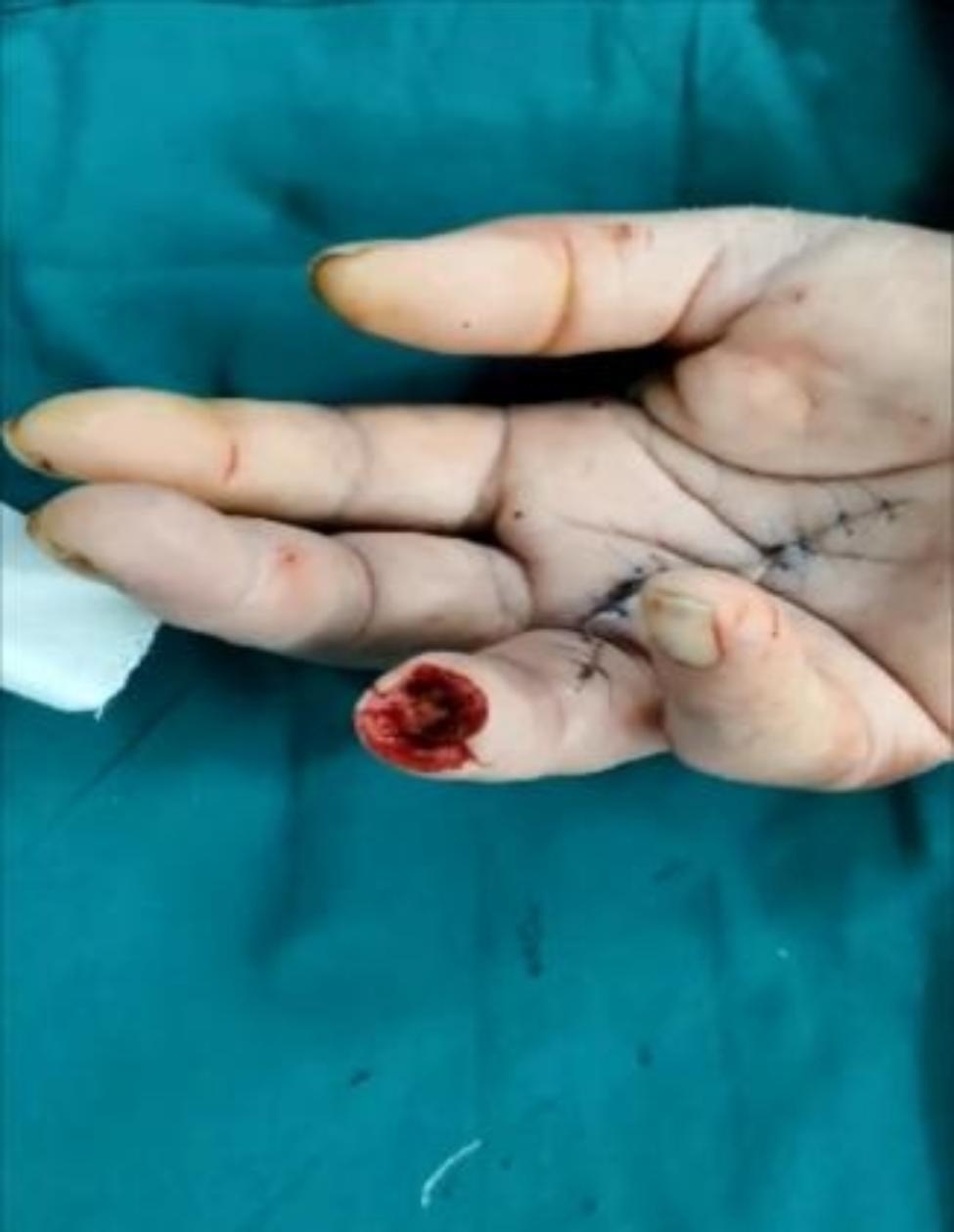
Good wound hygiene after debridement

A patient with RS in the right hand presented with a variation in the course of the common digital nerve and the corresponding artery; the course was divided into two branches, leading to the passage of the common digital artery. This 53-year-old female farmer presented with discoloration of both hands because of exposure to cold conditions for 2 years, which got aggravated 2 months back. The patient reported discoloration of both hands without obvious inducement, especially in the left hand, which was accompanied by pain and discomfort in both hands and feet. Furthermore, these symptoms worsened at night, followed by intermittent dull pain (Figs. 1, 2, 3, 4, 5, 6 and 7).

**Fig. 8:**
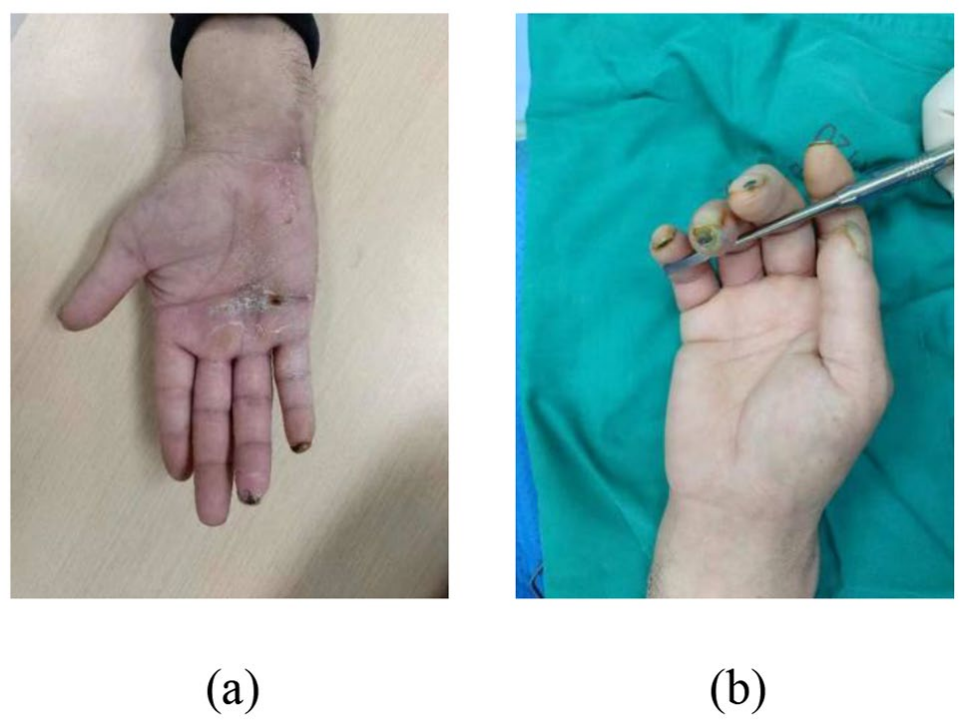
Preoperative exterior image

**Fig. 9:**
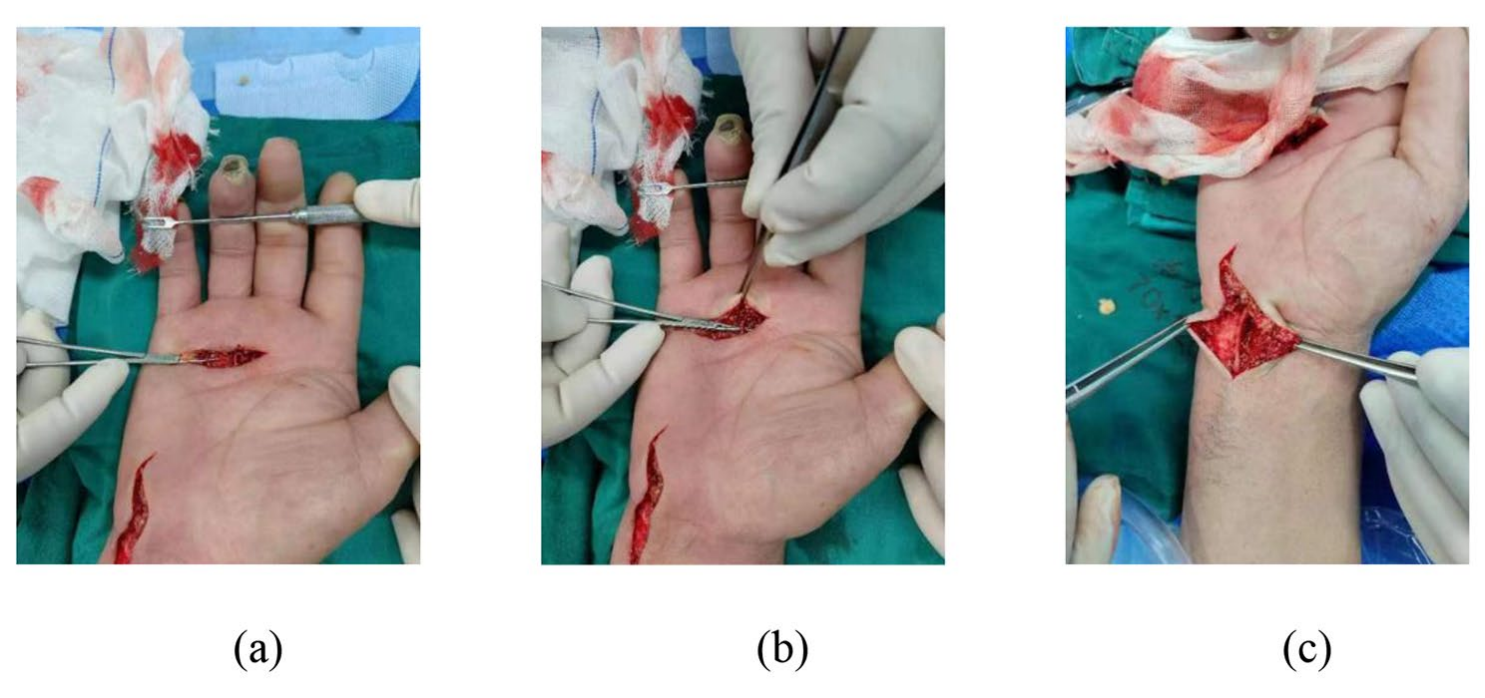
Surgical incision and the adventitia of blood vessels was stripped under the microscope

**Fig. 10:**
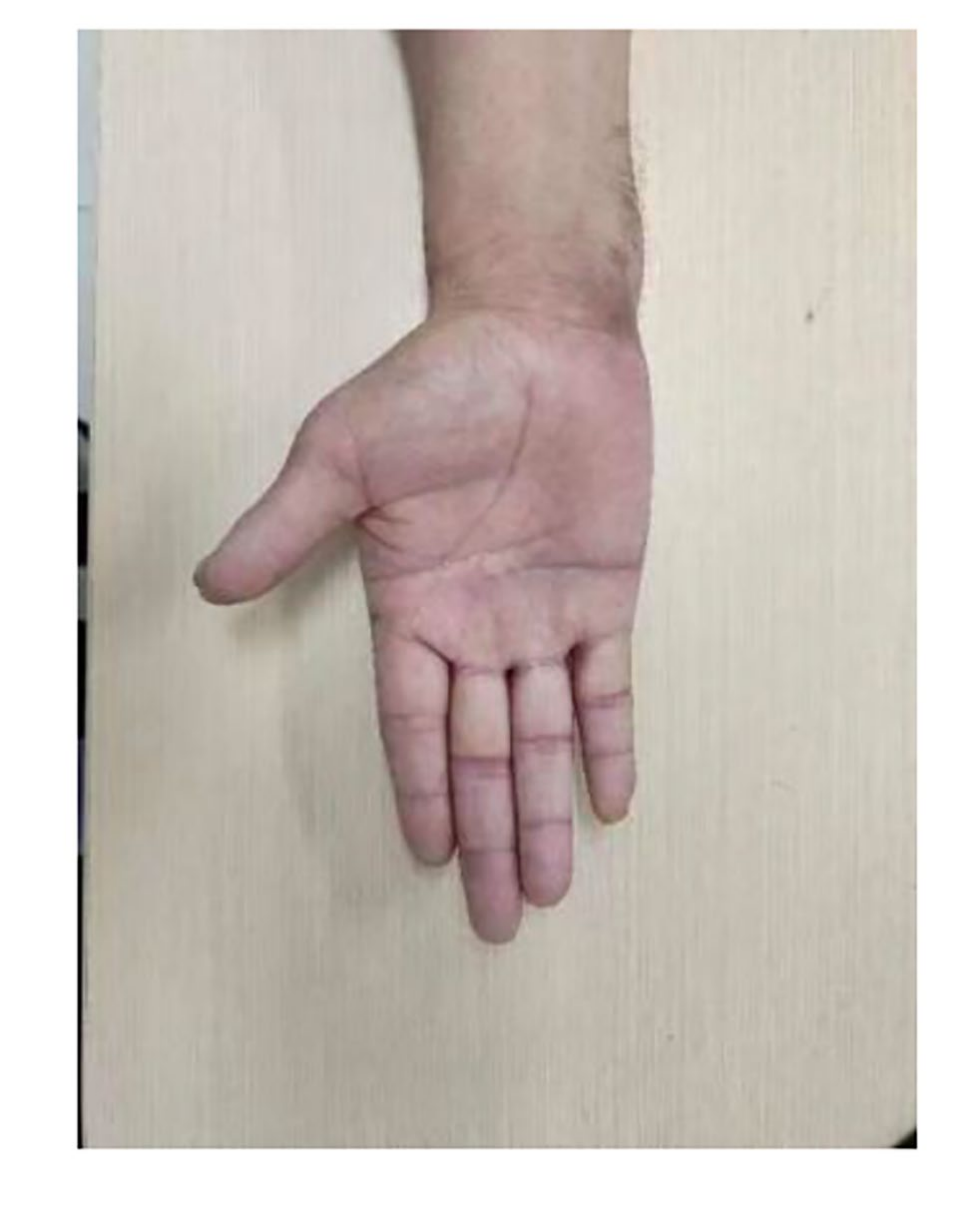
Postoperative outcomes, the blood flow of the patient’s fingers was unobstructed

The other case (Figs. 8, 9 and 10).

## Discussion

RS manifests as a transient spasmodic contractile response of acral vessels to cold or stressful stimuli. When this is solely driven by vasospasm absent of an underlying primary disease, it is termed primary RS. Crucially, our results have shed light on significant variations between our experimental and control groups. The “Mean Duration of RP attack” markedly differentiated the two cohorts, with the experimental group registering at 9.47 min ± 0.31 as opposed to 19.33 min ± 1.79 in the control group. Additionally, the RS Severity Scores were distinguishably different, clocking 8.55 for the experimental group and 11.23 for the control. Such statistical disparities not only validate our methodology but also underscore the distinct clinical presentations evident between the groups. Raynaud’s syndrome (RS), prevalent among young women, manifests as color changes in extremities in response to stimuli, with primary RS (PRP) being idiopathic and secondary RS (SRP) associated with autoimmune diseases like SLE and SSc. While its exact prevalence remains uncertain, primary and secondary RS affect 3-5% and 0.2% of the population, respectively. Its pathogenesis encompasses vascular, endovascular, and neurological facets. In PRP, there’s a disparity between vasoactive mediators, whereas SRP exhibits structural abnormalities like vascular intimal fibrosis. Notably, serological markers, such as EMP, VEGF, and Ang-II, indicate endothelial injuries in RS, emphasizing their diagnostic potential [11–14].

Treatment strategies prioritize symptom prevention, with biofeedback techniques benefiting many. However, pharmacotherapy, using drugs like nifedipine, remains effective in only 40-60% of patients. Surgical interventions, once reliant on thoracic sympathectomy, have transitioned towards minimally invasive approaches. Contemporary procedures, especially for severe RS manifestations, now lean on microscopic digital sympathectomy, underscoring the trend towards less invasive surgical solutions for RS [15–19].

Raynaud’s syndrome (RS) treatment emphasizes avoiding adverse stimuli. Biofeedback, beneficial in 92% of patients, aids in autonomic control. However, long-term symptom control is challenging. Pharmacotherapy, especially calcium channel blockers like nifedipine and diltiazem, benefits 40-60% of RS patients. Nifedipine decreases RS attack severity in 66%, whereas diltiazem serves as an alternative with fewer side effects.

Historically, thoracic sympathectomy was a surgical option for RS, but its efficacy waned over time. Modern strategies favor minimally invasive techniques, including microscopic periarterial sympathectomy with adventitial release. This method alleviates symptoms by facilitating sympathetic denervation of blood vessels. Techniques like periarterial sympathectomy have shown promise in both hands and feet. For instance, post-operative findings after metacarpophalangeal artery adventitial stripping in 84% of patients highlighted significant symptom relief and amputation prevention.

Another contemporary approach is the chemical sympathectomy using botulinum toxin injections near digital arteries. While it presents challenges, including dosage standardization and complications like muscle weakness, it has potential for symptom relief. Direct arterial bypass offers a remedy for ischemic RS cases with vessel occlusion, especially those beyond vasculitic arteriosclerosis. Recent studies, such as Liang et al.‘s work on adventitial stripping of the brachial artery, demonstrated an 81.8% complete relief rate without complications [20–33, 22].

As for the special case of variation in the vascular course found in this study, after a review of the literature, the authors found few relevant studies on such variations. In 2007, Tiandong Cheng and Qiuhong Tian from the Department of Clinical Medicine of North Sichuan Medical College and the Department of Human Anatomy of North Sichuan Medical College reported a case showing variation in the common palmar digital nerve. During an autopsy class on an adult male cadaver of yellow race in Nanchong (Sichuan, China), a variation was observed in which the common palmar digital nerve of the three branches of the right hand had clamped the corresponding common palmar digital artery. After setting the line from the scaphoid tubercle to the proximal pisiform bone as the reference line, the three common palmar digital nerves of this cadaveric specimen covered a distance of 5.312 cm, 5.470 cm, and 5.446 cm from the reference line, respectively. Furthermore, they were divided into two branches and clamped the corresponding common palmar digital artery, which was 6.560 cm, 6.776 cm, and 5.880 cm away from the distal reference line. Subsequently, the two branches converged into the common palmar digital nerve and traveled downwards near the metacarpal bone and were further divided into the proper palmar digital nerve that traveled along the entire finger’s margin till the fingertip. In our case, the common palmar digital artery’s diameter at the impression was 0.138 cm, 0.118 cm, and 0.126 cm in the first, second, and third fingers, respectively. Compared with the normal vascular diameter value, the diameters of all three arteries at the impression were smaller than that of the normal common palmar digital artery [34]. However, the course of the common palmar digital artery is similar to that of the common palmar digital nerve. Since this case report was similar to our study case, more attention should be paid to the presence of this variation in clinical palmar vascular nerve tendon exploration and repair, replantation of injured fingers and palms, vascular ligation, and surgical treatment of RS.

Additionally, it is paramount to emphasize the integrated perioperative and postoperative medical management for patients with severe vascular disease and ischemic digital ulcers undergoing periarterial sympathectomy. Beyond symptomatic relief achieved through analgesics, there is an imperative need for continuous vasculoprotection and blood flow preservation. This includes the administration of vasoactive and antithrombotic drugs, such as nifedipine in its retard release form, prostanoids, and antiplatelet agents. These pharmacological interventions ensure the maintenance of microvascular patency, thereby complementing the surgical intervention and preventing possible ischemic complications. The holistic approach of combining both surgical and pharmacological interventions underscores the necessity of a comprehensive treatment paradigm, focusing on both symptom alleviation and the underlying vascular pathophysiology.

While our study, with its short follow-up duration, underscores the safety and early effectiveness of the microsurgical techniques in RS patients, it is essential to recognize that a longer-term follow-up is imperative to assess the sustained benefits of this treatment approach. The primary insight from our study is that the minimally invasive treatment showcases reliable postoperative efficacy in the short term, circumventing complications associated with traditional thoracic and lumbar sympathectomy and bridging the gap posed by the limitations of simple drug treatments. As the surgical landscape evolves towards minimally invasive and refined operations, our study offers preliminary evidence in favor of microsurgery for RS patients. Yet, the study is limited by its retrospective nature and small sample size. Future research should thus be directed towards long-term, prospective cohort analysis to truly determine the enduring efficacy of the aforementioned surgical techniques for RS patients.

## Conclusion

Our study on a limited number of RS patients affirms the early effectiveness and safety of microsurgical techniques. This minimally invasive approach circumvents complications tied to traditional sympathectomy and addresses limitations of drug-only treatments. As surgical trends gravitate towards refined, minimally invasive procedures, our findings align with this trajectory. However, the study’s retrospective nature and scale impose constraints. Future endeavors should prioritize long-term, prospective analyses to comprehensively gauge the efficacy of digital sympathectomy alongside digital artery’s adventitial release. Notably, our insights underscore the immediate benefits of microsurgery for RS, but extended follow-ups remain paramount for definitive conclusions.

## Data Availability

The data presented in this study are available on request from the corresponding author.

## List of abbreviations

EMP: Endothelial microparticle
RS: Raynaud’s syndrome
VEGF: Vascular endothelial growth factor, f

## References

[1] Ruaro B, Smith V, Sulli A, Pizzorni C, Tardito S, Patané M (2019). Innovations in the Assessment of primary and secondary Raynaud’s Phenomenon. Front Pharmacol.

[2] Herrick AL (2019). Raynaud’s phenomenon. J Scleroderma Relat Disord.

[3] Flavahan NA (2015). A vascular mechanistic approach to understanding Raynaud phenomenon. Nat Rev Rheumatol.

[4] Chikura B, Moore TL, Manning JB, Vail A, Herrick AL (2008). Sparing of the thumb in Raynaud’s phenomenon. Rheumatology (Oxford).

[5] Coffman JD, Cohen AS (1971). Total and capillary fingertip blood flow in Raynaud’s phenomenon. N Engl J Med.

[6] Roustit M, Blaise S, Allanore Y, Carpentier PH, Caglayan E, Cracowski JL (2013). Phosphodiesterase-5 inhibitors for the treatment of secondary Raynaud’s phenomenon: systematic review and meta-analysis of randomised trials. Ann Rheum Dis.

[7] Iorio ML, Masden DL, Higgins JP (2012). Botulinum toxin A treatment of Raynaud’s phenomenon: a review. Semin Arthritis Rheum.

[8] Momeni A, Sorice SC, Valenzuela A, Fiorentino DF, Chung L, Chang J (2015). Surgical treatment of systemic sclerosis–is it justified to offer peripheral sympathectomy earlier in the Disease process?. Microsurgery.

[9] Bello RJ, Cooney CM, Melamed E, Follmar K, Yenokyan G, LeatHerman G (2017). The therapeutic efficacy of botulinum toxin in treating scleroderma-associated raynaud’s phenomenon: a randomized, double-blind, placebo-controlled clinical trial. Arthritis Rheumatol.

[10] Daniels J, Pauling JD, Eccleston C (2018). Behaviour change interventions for the management of Raynaud’s phenomenon: a systematic literature review. BMJ Open.

[11] Pope JE (2007). The diagnosis and treatment of Raynaud’s phenomenon: a practical approach. Drugs.

[12] Valdovinos ST, Landry GJ (2014). Raynaud syndrome. Tech Vasc Interv Radiol.

[13] Freedman RR, Mayes MD (1996). Familial aggregation of primary Raynaud’s Disease. Arthritis Rheum.

[14] Coffman JD (1991). Raynaud’s phenomenon. An update. Hypertension.

[15] Herrick AL (2005). Pathogenesis of Raynaud’s phenomenon. Rheumatology (Oxford).

[16] Hasegawa M, Nagai Y, Tamura A, Ishikawa O (2006). Arteriographic evaluation of vascular changes of the extremities in patients with systemic sclerosis. Br J Dermatol.

[17] Jung C, Drummer K, Oelzner P, Figulla HR, Boettcher J, Franz M (2015). The association between endothelial microparticles and inflammation in patients with systemic sclerosis and Raynaud’s phenomenon as detected by functional imaging. Clin Hemorheol Microcirc.

[18] Manetti M, Guiducci S, Romano E, Ceccarelli C, Bellando-Randone S, Conforti ML (2011). Overexpression of VEGF165b, an inhibitory splice variant of vascular endothelial growth factor, leads to insufficient angiogenesis in patients with systemic sclerosis. Circ Res.

[19] Edwards CM, Marshall JM, Pugh M (1998). Lack of habituation of the pattern of cardiovascular response evoked by sound in subjects with primary Raynaud’s Disease. Clin Sci (Lond).

[20] Freedman RR (1991). Physiological mechanisms of temperature biofeedback. Biofeedback Self Regul.

[21] Raynaud’s Treatment Study Investigators (2000). Comparison of sustained-release nifedipine and temperature biofeedback for treatment of primary Raynaud phenomenon. Results from a randomized clinical trial with 1-year follow-up. Arch Intern Med.

[22] Coveliers HM, Hoexum F, Nederhoed JH, Wisselink W, Rauwerda JA (2011). Thoracic sympathectomy for digital ischemia: a summary of evidence. J Vasc Surg.

[23] Dong G, Zhang N, Zhao J (2001). Sympathetic ganglionectomy for the treatment of Raynaud’s Disease. Chin J Minimally Invasive Surg.

[24] O’Connor KJ, Grady JF, Moore CJ, Axe TM, Shumaker JM (1996). Hallux amputation in combination with a lumbar sympathectomy for treatment of a non-healing ulceration in a patient with Buerger’s Disease. J Foot Ankle Surg.

[25] O’Brien B, Kumar PAV, Mellow CG, Oliver TV (1992). Radical microarteriolysis in the treatment of vasospastic disorders of the hand, especially scleroderma. J Hand Surg Eur Vol.

[26] Yee AM, Hotchkiss RN, Paget SA (1998). Adventitial stripping: a digit saving procedure in refractory Raynaud’s phenomenon. J Rheumatol.

[27] WasSerman A, Brahn E (2010). Systemic sclerosis: bilateral improvement of Raynaud’s phenomenon with unilateral digital sympathectomy. Semin Arthritis Rheum.

[28] Balogh B, Mayer W, Vesely M, Mayer S, Partsch H, Piza-Katzer H (2002). Adventitial stripping of the radial and ulnar arteries in Raynaud’s Disease. J Hand Surg Am.

[29] Kotsis SV, Chung KC (2003). A systematic review of the outcomes of digital sympathectomy for treatment of chronic digital ischemia. J Rheumatol.

[30] Gofeld M, Faclier G (2006). Bilateral pain relief after unilateral thoracic percutaneous sympathectomy. Can J Anaesth.

[31] Han KR, Kim C, Park EJ (2008). Successful treatment of digital ulcers in a scleroderma patient with continuous bilateral thoracic sympathetic block. Pain Physician.

[32] Mannava S, Plate JF, Stone AV, Smith TL, Smith BP, Koman LA (2011). Recent advances for the management of Raynaud phenomenon using botulinum neurotoxin A. J Hand Surg Am.

[33] Liang N, Guan Q, Liu Z (2003). Clinical experience in treating 11 cases of Raynaud’s Disease with exfoliation of brachial artery. Shanxi Med J.

[34] Tian D, Tian Q, Xie X, Wang J, Dai X, Chen H (2007). Variation of palmar digital nerve: a case report. J North Sichuan Med Coll.

