# Correction: Efficacy analysis of minimally invasive surgery for Raynaud’s syndrome

**DOI:** 10.1186/s12893-023-02265-3

**Published:** 2023-11-16

**Authors:** Fengwei Yu, Yongtao Liu, Chengnian Zhang, Botao Pang, Daijie Zhang, Wei Zhao, Xuecheng Li, Weiqiang Yang

**Affiliations:** 1 Hand Microsurgery, Binzhou Medical University Hospital, Binzhou, 256600 China; 2 The First Clinical School of Binzhou Medical University, Binzhou, 256600 China

**Correction to: BMC Surgery (2023) 23:1**

10.1186/s12893-023-02225-x

In the original version of this article [1], author “Fengwei Yu and Chengnian Zhang” should need to be denoted as an equally contributing author.

The original article has been corrected.
